# Supplementary material for: Pyruvate Dehydrogenase Kinase 1 inhibition mediated oxidative phosphorylation enhancement in cartilage promotes osteoarthritis progression
Source: BMC Musculoskelet Disord. 2023 Jul 20;24:597. doi: 10.1186/s12891-023-06585-6 (PMC10357736; doi:10.1186/s12891-023-06585-6)
Supplement: Supplementary file 1 — Additional file 1. [file 12891_2023_6585_MOESM1_ESM.zip › BP.Gsea.1653623667859/GOBP_PROTEIN_TARGETING_TO_PEROXISOME.html]

Details for gene set GOBP\_PROTEIN\_TARGETING\_TO\_PEROXISOME[GSEA]

|  || Dataset | 55457\_collapsed\_to\_symbols.cls.cls #OA\_versus\_normal.cls.cls #OA\_versus\_normal\_repos |
| Phenotype | cls.cls#OA\_versus\_normal\_repos |
| Upregulated in class | OA |
| GeneSet | GOBP\_PROTEIN\_TARGETING\_TO\_PEROXISOME |
| Enrichment Score (ES) | 0.4808909 |
| Normalized Enrichment Score (NES) | 1.5176672 |
| Nominal p-value | 0.05159705 |
| FDR q-value | 0.35508397 |
| FWER p-Value | 1.0 |
Table: GSEA Results Summary

  

Fig 1: Enrichment plot: GOBP\_PROTEIN\_TARGETING\_TO\_PEROXISOME      
 Profile of the Running ES Score & Positions of GeneSet Members on the Rank Ordered List

  

| SYMBOL | TITLE | RANK IN GENE LIST | RANK METRIC SCORE | RUNNING ES | CORE ENRICHMENT || 1 | PEX3 | peroxisomal biogenesis factor 3 [Source:HGNC Symbol;Acc:HGNC:8858] | 11 | 1.178 | 0.1614 | Yes |
| 2 | PEX12 | peroxisomal biogenesis factor 12 [Source:HGNC Symbol;Acc:HGNC:8854] | 74 | 0.956 | 0.2882 | Yes |
| 3 | PEX2 | peroxisomal biogenesis factor 2 [Source:HGNC Symbol;Acc:HGNC:9717] | 94 | 0.921 | 0.4136 | Yes |
| 4 | PEX7 | peroxisomal biogenesis factor 7 [Source:HGNC Symbol;Acc:HGNC:8860] | 467 | 0.673 | 0.4775 | Yes |
| 5 | PEX10 | peroxisomal biogenesis factor 10 [Source:HGNC Symbol;Acc:HGNC:8851] | 1253 | 0.467 | 0.4809 | Yes |
| 6 | PEX19 | peroxisomal biogenesis factor 19 [Source:HGNC Symbol;Acc:HGNC:9713] | 2223 | 0.336 | 0.4520 | No |
| 7 | PEX1 | peroxisomal biogenesis factor 1 [Source:HGNC Symbol;Acc:HGNC:8850] | 3655 | 0.190 | 0.3671 | No |
| 8 | PEX14 | peroxisomal biogenesis factor 14 [Source:HGNC Symbol;Acc:HGNC:8856] | 3985 | 0.157 | 0.3631 | No |
| 9 | PEX13 | peroxisomal biogenesis factor 13 [Source:HGNC Symbol;Acc:HGNC:8855] | 6228 | -0.023 | 0.1923 | No |
| 10 | PEX6 | peroxisomal biogenesis factor 6 [Source:HGNC Symbol;Acc:HGNC:8859] | 6325 | -0.030 | 0.1890 | No |
| 11 | ZFAND6 | zinc finger AN1-type containing 6 [Source:HGNC Symbol;Acc:HGNC:30164] | 6911 | -0.073 | 0.1536 | No |
| 12 | PEX16 | peroxisomal biogenesis factor 16 [Source:HGNC Symbol;Acc:HGNC:8857] | 8921 | -0.226 | 0.0288 | No |
| 13 | RAB8B | "RAB8B, member RAS oncogene family [Source:HGNC Symbol;Acc:HGNC:30273]" | 10223 | -0.343 | -0.0249 | No |
| 14 | PEX26 | peroxisomal biogenesis factor 26 [Source:HGNC Symbol;Acc:HGNC:22965] | 10303 | -0.351 | 0.0173 | No |
| 15 | PEX5 | peroxisomal biogenesis factor 5 [Source:HGNC Symbol;Acc:HGNC:9719] | 10737 | -0.398 | 0.0385 | No |
| 16 | PEX5L | peroxisomal biogenesis factor 5 like [Source:HGNC Symbol;Acc:HGNC:30024] | 10805 | -0.406 | 0.0892 | No |
| 17 | LONP2 | "lon peptidase 2, peroxisomal [Source:HGNC Symbol;Acc:HGNC:20598]" | 11699 | -0.532 | 0.0932 | No |
Table: GSEA details [plain text format]

  

Fig 2: GOBP\_PROTEIN\_TARGETING\_TO\_PEROXISOME      
 Blue-Pink O' Gram in the Space of the Analyzed GeneSet

  

Fig 3: GOBP\_PROTEIN\_TARGETING\_TO\_PEROXISOME: Random ES distribution      
 Gene set null distribution of ES for **GOBP\_PROTEIN\_TARGETING\_TO\_PEROXISOME**
